# Ultrasonography reference values for the calcaneus in children and adolescents living at high altitude in Peru

**DOI:** 10.3389/fendo.2025.1490086

**Published:** 2025-02-21

**Authors:** Jose Fuentes-López, Rubén Vidal-Espinoza, Ofelia Mamani-Luque, Vladimiro Ibanez-Quispe, Claudia Villegas-Abrill, Bernabé Canqui-Flores, Charles Ignacio Mendoza-Mollocondo, Lucila Sanchez-Macedo, Marco Cossio-Bolaños, Rossana Gomez-Campos

**Affiliations:** ^1^ Universidad Nacional del Altiplano, Puno, Peru; ^2^ Universidad Católica Silva Henríquez, Santiago, Chile; ^3^ Universidad San Ignacio de Loyola, Lima, Peru

**Keywords:** bone health, children, quantitative ultrasound, percentiles, QUS measurement

## Abstract

**Objective:**

The evaluation of bone health during the growth stage is extremely important, as it is a key factor to prevent bone diseases in adulthood. The objectives of the study were: a) to verify if there are differences in bone health with other geographic regions, b) to develop bone health curves using quantitative ultrasonography (QUS) through the Broadband Ultrasonic Attenuation (BUA) parameter in children and adolescents residing in a high altitude region of Peru and c) to determine specific cut-off points for bone health assessment in this particular population.

**Methods:**

A cross-sectional study was carried out in schoolchildren in a high altitude region of Peru. The sample consisted of 1468 children and adolescents (724 males and 744 females). The age range was 6.0 to 17.9 years old. Weight and height were evaluated. Body Mass Index (BMI) was calculated. Bone quality was evaluated by quantitative ultrasonography (QUS) of the calcaneus. The parameters measured were Speed of Sound (SOS, m/s); Broadband Ultrasonic Attenuation (BUA, dB/MHz); and Bone Quality Index (BQI= αSOS+αBUA, αβ: temperature corrections).

**Results:**

There were small discrepancies in bone health (BUA) between studies from various geographic regions. Values differed across all age ranges from ~0.36 to ~10.86 in males and from ~0.26 to ~6.68 in females. At later ages during adolescence the values are relatively similar, reaching a plateau around 16 and 17 years of age. Percentiles were calculated for BUA by age and sex. Sensitivity and specificity values in females are slightly higher relative to males. However, the Youden Index reflects 0.84 for both sexes and the appropriate cut-off point for men is ≤67.8 and for women is ≤63.7.

**Conclusion:**

The study demonstrated that there are small discrepancies in bone health (BUA) among children between children and adolescents from different geographic regions. These findings support the creation of specific references and cut-off points for bone health in the pediatric population of a high altitude region of Peru. The results suggest the application of percentiles for the assessment of bone health in school and epidemiological contexts.

## Introduction

Bone health is defined as bone resistance to fracture, determined by the assessment of bone mineral reserve, expressed as bone mineral content (BMC) or bone mineral density (BMD) (1). Its assessment is extremely important during all stages of life, as it serves to understand early precursors of bone fractures and diseases such as osteopenia and osteoporosis (2, 3).

In recent years, the assessment of bone health during the growth and development stage has gained increased relevance, due to the recognition of the importance of good bone density in childhood and adolescence as a key factor in preventing bone diseases in adulthood (4, 5).

In fact, during the growth period, the skeleton undergoes constant changes involving both bone modeling and remodeling, crucial processes for the formation of a robust and functional skeleton (1). For during these periods, the foundation is laid for bone and muscle health that will last throughout life.

The “bone bank” is built in the first two decades of life, and most of the risk of osteoporosis depends on what happens in this period (6). This highlights the importance of adopting healthy habits, such as proper nutrition and regular physical activity, to maximize skeletal and muscular development (7).

Consequently, given the growing interest in studying osteoporosis at pediatric ages (8, 9). An interest in developing bone health assessment curves in children and adolescents has emerged in recent years (10–12). This approach has driven the use of advanced technologies for bone density measurement, highlighting among current clinical diagnostic methods the use of dual-energy X-ray absorptiometry (DXA) and quantitative ultrasound (QUS) as main tools (5, 13, 14).

In this context, quantitative ultrasonography QUS is a relatively inexpensive and non-invasive method that assesses bone status in various populations (15–17). It presents three measurement parameters (Speed of Sound (SOS, m/s); Broadband Ultrasonic Attenuation (BUA, dB/MHz); and Bone Quality Index (BQI= αSOS+αBUA, αβ: temperature corrections).

In fact, studies have evidenced that calcaneal BUA shows better correlation with BMD and BMC (18–20). In addition, BUA measured by QUS technology has been shown to predict fracture risk in certain populations. Perhaps even with better predictive power than DXA (19–21) and offers an option for bone health assessment in resource-poor regions and settings (20).

In this context, several studies have developed bone health curves using quantitative ultrasonography (QUS) and the Broadband Ultrasonic Attenuation (BUA) parameter in children and adolescents from various geographic regions of the world (13, 15, 16, 22, 23). Including a study conducted at moderate altitude (17). However, to date, similar studies have not been conducted in high-altitude regions, where environmental conditions could have a significant impact on bone development.

Children living at high altitudes often experience various endocrine and metabolic conditions due to hypoxia. For example, children and adolescents living at high altitude often show a small delay in linear growth and skeletal maturation (24). In addition, at high altitudes energy expenditure has shown a higher basal metabolic rate over energy expenditure (25, 26). This is due to unintentional physical activity when hiking in steep terrain (27). Even, due to low economic income in these populations, lower prevalence of overweight and obesity has also been observed respectively (28).

This lack of data underscores the need to investigate how these parameters behave in pediatric populations living at high altitudes in Peru (3820 meters above sea level). Considering that the existing bone health curves may not be applicable in these specific contexts.

Therefore, this study aims to: a) verify if there are differences in bone health with other geographic regions, b) develop bone health curves using quantitative ultrasonography (QUS) through the Broadband Ultrasonic Attenuation (BUA) parameter in children and adolescents residing in a high altitude region of Peru and c) determine specific cut-off points for bone health assessment in this particular population.

## Materials and methods

### Type of study and sample

A cross-sectional study was carried out in schoolchildren in a high altitude region of Peru. The sample consisted of 1468 children and adolescents (724 males and 744 females). The age range was 6.0 to 17.9 years old. The schoolchildren belonged to public schools in the city of Puno, Peru. The sample selection was non-probabilistic (accidental). The city of Puno is located at 3820 meters above sea level and borders with La Paz (Bolivia).

Schoolchildren who regularly attended each of the schools were included in the study. Those who regularly attended each of the schools. Who attended physical education classes (once a week). In addition, those who completed the anthropometric and calcaneal ultrasonography evaluations.

Schoolchildren who did not authorize participation in the study and those who had any impairment and/or physical limitation that prevented the evaluations were excluded.

Parents gave written informed consent and the children and adolescents gave their assent to participate in the evaluations in their schools. The study was conducted according to the Helsinki declaration for human beings and according to the ethics committee of the Universidad Nacional del Altiplano, Puno (007-2022).

### Techniques and procedures

A team of 4 physical education professionals with extensive experience in anthropometric and bone health evaluations (ultrasonography) was formed. The team went to each of the schools to carry out the evaluations. This procedure was carried out from April to October 2023.

Anthropometric measurements were evaluated according to the suggestions described by Ross and Marfell-Jones (29). Weight and height were assessed with as little clothing as possible (barefoot, shorts and T-shirt). A Tanita digital scale (United Kingdom, Ltd.) with an accuracy of 0.1 kg and a range of 0.1 kg to 150 kg was used. A portable stadiometer (Hamburg, Seca, Ltd.) with an accuracy of 0.1 mm and a measuring range of 0.0 cm to 220.0 cm. Body mass index (BMI) was calculated. BMI = weight (kg)/height (m)^2^]. To verify the reliability of the anthropometric measurements, the evaluations were performed twice. The relative Technical Measurement Error (TEM%) intra-evaluator was less than 0.85%.

QUS data collection was performed using a SONOST 3000 bone densitometer (Seoul, South Korea). The parameters measured were Speed of Sound (SOS, m/s); Broadband Ultrasonic Attenuation (BUA, dB/MHz); and Bone Quality Index (BQI= αSOS+αBUA, αβ: temperature corrections). The volunteer was seated and barefoot on the right foot. For the measurement, gel was passed to the right foot, then placed inside the measurement chamber. The evaluation of each subject lasted approximately 15 to 20seconds. Ten percent (147 subjects) of the sample was evaluated twice. The TEM% intra-evaluator was less than 1%. The 4 evaluators were trained by the manufacturer to evaluate, analyze and interpret information on bone quality and fracture risk. Through the measurement of sound velocity and ultrasound attenuation by bandwidth.

### Statistics

Statistical analysis was performed in the SPSS 18.0 statistical program. The data set was subjected to the Kolmogorov-Smirnov K-S normality test. The descriptive statistics of arithmetic mean, standard deviation and range were analyzed. The difference between both sexes was verified by t-test for independent samples. The BUA values were used to construct the percentile distribution by means of the LMS technique: L (Lambda; skewness), M (Mu; median) and S (Sigma; coefficient of variation) (30). The percentiles calculated were: P3, P5, P10, P15, P25, P50, P75, P85, P90, P95 and P97. Calculations were performed using LMS Chart Maker version 2.3 software (31). The area under the curve (AUC) receiver operating characteristic (ROC) was calculated to evaluate the performance of a classification model. In addition, we determined sensitivity and specificity values, as well as the Youden Index to assess the performance of the classification model, in order to identify the optimal threshold that maximizes both sensitivity and specificity. The significance of p<0.05 was adopted in all calculations.

## Results

The variables that characterize the studied sample of high altitude schoolchildren in Peru are shown in **Table 1**. Males presented greater body weight than females from 15 to 17 years of age (p<0.05). In height, there were significant differences, with males presenting greater height than females from 13 to 17 years of age. In BMI, males presented higher BMI than females from 15 to 17 years of age. In the SOS, males presented higher values than females from 12 to 17 years of age. In the BUA, males presented higher values at 7 years of age. Meanwhile, at 13, 14 and 17 years, males presented significantly higher values than females (p>0.05).

**Table 1:** Anthropometric and ultrasonography characteristics of the studied sample.

Age (years)	n	Weight (kg)		Height (cm)		BMI (kg/m^2^)		SOS (m/s)		BUA (dB/MHz)		BQI (SOS+βBUA)	
		X	SD	X	SD	X	SD	X	SD	X	SD	X	SD
Males													
6	59	24.5	6.4	119.6	6.6	17.1	4	1494.1	11.4	68.2	19	58.7	12.1
7	56	26.1	5	122.9	6.4	17.2	2.5	1495.6	12.5	67.1	15.3	59.3	10.2
8	49	30.7	6.5	128.5	5	18.5	3.1	1495.3	10.5	67.7	15.9	59.4	10.6
9	52	34.1	8.5	133.2	6.3	19.1	3.8	1494.9	10.8	70	18.7	60	11.1
10	60	37.5	9	138.1	6	19.4	3.9	1494.7	11	69.4	13.9	59.6	8.5
11	52	41.6	9.6	143.9	7	20	3.8	1496.1	9.6	73.7	12.7	62.4	10.4
12	67	46.6	10.7	150.7	6.8	20.4	4	1501.4*	11.5	76.9	12.9	67.5	10
13	70	52	10.6	157.9*	6.5	20.8	3.8	1502.2*	10.6	78	12.8	68.5	11.3
14	68	55.8	11.2	163.8*	6.2	20.7	3.4	1504.4*	12	83.4	15.6	72.3	13.3
15	70	58.9*	10.2	166.6*	5.2	21.2*	3.4	1503.6*	11.6	86.4	15.7	73	13.4
16	60	62.2*	13.2	167.9*	5.4	22.1*	4.6	1507.5*	11.1	93	18.2	78.6	13.6
17	61	62.8*	9.8	167.8*	5.6	21.9*	3.3	1507.3*	13.9	94.5	18.8	79	15.7
Females													
6	44	22.7	8	116.2	3.9	16.8	5.9	1492.4	7.7	66	9.6	56.6	7
7	91	25.4	7.5	121.6	5.3	17.2	4.7	1493.6	8.9	60.9	8.7	55.3	6.5
8	80	28.1	5.4	127.2	5.3	17.3	2.6	1493.7	7.3	64.7	10.6	57	7.1
9	98	32.4	8.2	132.6	6.5	18.3	3.7	1492.3	8.5	66.2	11.1	56.6	8.3
10	98	37.1	6.7	140.6	6	18.7	2.9	1495.7	8.8	71.5	13.5	61.2	9.5
11	77	41.5	9.7	145.3	6.5	19.5	3.5	1497.4	10	75.6	12.5	64.1	11.1
12	55	45.7	9	148.3	6.4	20.6	3.1	1493.1	9.1	78	9.5	62	9.2
13	38	49.3	7.7	151	4.9	21.5	2.6	1492.5	9.1	84.5	12.6	64.3	10
14	49	54.5	8.6	155.1	4.5	22.7	3.4	1496.4	11.1	89.3	11.4	69	10.8
15	39	54.5	7.5	152.9	4.3	23.3	2.9	1502.2	10.8	89.6	10.6	73.3	10.1
16	38	58.5	11.5	155.8	4.1	24.1	4.4	1505.5	15.7	92.1	14.7	76.7	16.2
17	37	58.6	11.6	156	6.3	23.3	4.9	1503.6	12.3	87.6	12	73.5	11.5

X, Mean; SD, Standard deviation; SOS, Speed of sound; BUA, Broadband ultrasonic attenuation; BQI, αSOS+αBUA; *significant difference in relation to females.

**Figure 1:**
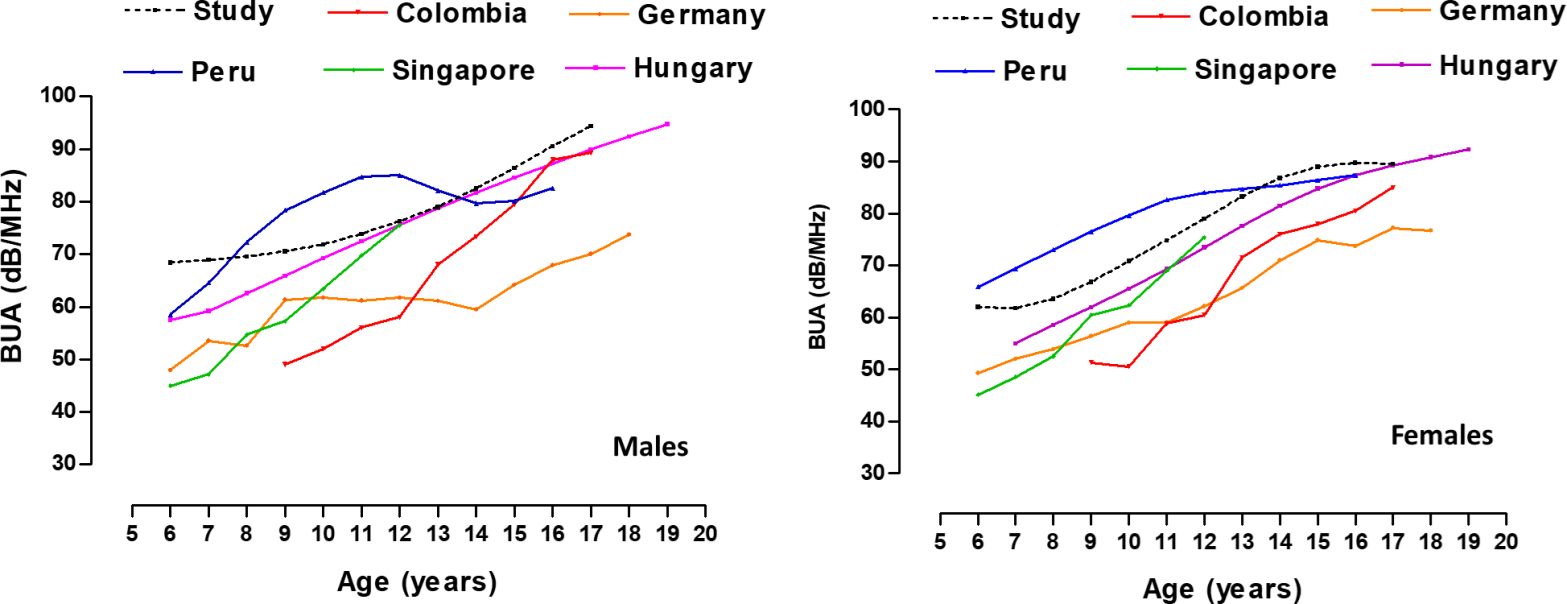
Comparison of BUA values (50th percentile) of children of adolescents from different geographic regions.

Comparisons between studies with other geographic regions are shown in **Table 2**. These comparisons show the mean ± SD values of the BUA of both sexes from 6 to 20 years of age. **Figure 1** shows the slight discrepancies between the study carried out at high altitude in Peru and other studies carried out in various regions of the world. Comparisons were made using the 50th percentile.

**Table 2:** Comparison of descriptive values (X $\bar{\pm }$ SD) of BUA for calcaneus in children and adolescents from various geographic regions.

N°	Authors	Year	Country	Subjects	range (age)		Age (years)												
							6	7	8	9	10	119	12	13	14	15	16	17	18
Males																			
1	Study	2024	Perú	724	6 a 17	X	68.17	67.1	67.74	69.96	69.38	73.74	76.94	77.96	83.4	86.35	93.04	94.46	
						SD	19	15.29	15.89	18.72	13.88	12.65	12.87	12.8	15.64	15.68	18.17	18.77	
2	Gómez-Campos et al [17]	2019	Perú	565	6 a 16	X	61.6	61.5	75.5	82.5	80.5	86.9	93.3	75.7	67.4	80.9	90.8		
						SD	11.7	14.2	16.9	18.3	10.7	10.4	16.0	15.4	11.1	6.2	9.8		
3	Ramirez et al [16]	2015	Colombia	445	9 a 17	X				48.6	52.9	57.2	60.6	68.2	73.9	81.2	87.6	90.2	
						SD				9.2	10.4	10.1	10.5	13.3	11.5	15.9	16.6	18.8	
4	Goh et al [32]	2011	Singapore	417	6 a 12	X	44.9	47.1	54.6	57.2	63.4	69.7	75.4						
						SD	10.9	8.7	10.5	10.5	12.7	14.4	13.1						
5	Wunsche et al [33]	2000	Germany	1676	6 a 18	X	47.9	53.5	56.2	61.3	61.7	61.2	61.7	61.1	59.5	64.1	67.9	70.0	73.7
						SD	9.3	10.4	9.8	11.1	11.3	10.7	11.4	13.2	12.1	14.0	14.8	13.6	13.3
6	Szmodis et al [15]	2017	Hungary	1349	7 a 19	X		54.9	64.4	64.8	67.9	75.0	77.3	78.9	78.9	86.7	89.4	95.2	82.7
						SD		16.5	12.8	12.3	9.7	11.9	12.3	9.8	10.6	14.3	14.4	16.5	17.1
Females																			
1	Study	2024	Perú	744	6 a 17	X	66.02	60.94	64.7	66.21	71.46	75.55	77.98	84.5	89.26	89.59	92.06	87.57	
						SD	9.61	8.66	10.56	11.06	13.52	12.5	9.46	12.58	11.41	10.55	14.71	12.02	
2	Gómez-Campos et al [17]	2019	Perú	611	6 a 16	X	67.8	71.4	72.8	79.6	75.9	87.1	85.4	70.9	71.8	91.5	87.9		
						SD	12.2	11.3	15.9	13.9	14.8	13.8	8.2	10.5	20.7	24.3	12.3		
3	Ramirez et al [16]	2015	Colombia	556	9 a 17	X				51.8	51.7	60.5	63.7	71.1	75.6	79.8	82.3	83.0	
						SD				8.9	10.6	13.3	14.4	10.4	11.8	13.0	14.8	15.0	
4	Goh et al [32]	2011	Singapore	333	6 a 12	X	45.1	48.5	52.5	60.4	62.2	68.9	75.4						
						SD	10.8	10.3	9.02	11.1	9.5	13.6	19.4						
5	Wunsche et al [33]	2000	Germany	1623	6 a 18	X	49.3	52.0	53.9	56.4	59.0	59.1	62.2	65.7	71.0	74.9	73.7	77.2	76.7
						SD	10.0	8.8	8.3	10.2	9.4	10.7	13.9	13.5	16.4	13.2	14.5	14.6	15.9
6	Szmodis et al [15]	2017	Hungary	1325	7 a 19	X		54.1	60.5	64.0	63.5	68.6	75.9	78.0	84.4	85.2	88.0	91.3	85.4
						SD		10.3	8.52	9.2	8.6	11.7	12.	14.3	13.2	10.6	14.2	14.4	17.6

X, Mean; SD, Standard deviation.

At early ages (from 6 to 12 years), the children in the (high altitude) and moderate altitude study from Peru (17) showed higher values than the other studies conducted in Colombia (16), Singapore (32), Germany (33) and Hungary (15). Overall, BUA discrepancies (dB/MHz) between studies ranged from ~0.36 to ~10.86 in males and from ~0.26 to ~6.68 in females. At later ages during adolescence the values are relatively similar, reaching a plateau around 16 and 17 years of age.

The distribution of BUA percentiles is shown in **Table 3**. In both sexes the p50 values increase with advancing age.

**Table 3:** Distribution of smoothed BUA percentiles by age and sex.

Age	Males													
	L	M	S	P3	P5	P10	P15	P25	P50	P75	P85	P90	P95	P97
Males														
6	1.092	68.403	0.223	39.06	42.83	48.58	52.42	58.05	68.4	78.61	84.04	87.7	93.1	96.59
7	1.225	68.847	0.217	39.1	43.06	49.00	52.92	58.59	68.85	78.77	83.97	87.46	92.57	95.85
8	1.332	69.488	0.211	39.48	43.59	49.68	53.65	59.34	69.49	79.17	84.2	87.56	92.45	95.58
9	1.372	70.504	0.205	40.67	44.78	50.86	54.81	60.46	70.5	80.04	84.98	88.27	93.06	96.13
10	1.335	71.849	0.2	42.66	46.63	52.54	56.39	61.94	71.85	81.32	86.25	89.54	94.34	97.41
11	1.227	73.841	0.194	45.51	49.25	54.88	58.61	64.02	73.84	83.37	88.38	91.74	96.66	99.83
12	1.081	76.251	0.189	48.76	52.26	57.63	61.22	66.5	76.25	85.9	91.04	94.51	99.63	102.95
13	0.918	78.997	0.183	52.21	55.52	60.64	64.12	69.29	79	88.81	94.11	97.71	103.07	106.57
14	0.767	82.488	0.178	56.02	59.21	64.2	67.61	72.73	82.49	92.53	98.02	101.78	107.42	111.12
15	0.64	86.354	0.173	59.98	63.09	68	71.39	76.5	86.35	96.63	102.31	106.23	112.13	116.02
16	0.525	90.489	0.168	64.09	67.16	72.03	75.41	80.53	90.49	101	106.87	110.93	117.09	121.17
17	0.416	94.34	0.163	68.01	71.04	75.85	79.21	84.32	94.34	105.03	111.04	115.22	121.59	125.83
Females														
6	1.174	62.013	0.172	41.2	43.9	48	50.7	54.7	62	69.2	72.9	75.5	79.2	81.6
7	1.007	61.699	0.168	42.2	44.7	48.4	51	54.7	61.7	68.7	72.4	74.9	78.7	81.1
8	0.786	63.567	0.163	44.7	47	50.6	53	56.7	63.6	70.6	74.5	77.2	81.1	83.7
9	0.514	66.813	0.159	48.3	50.5	53.9	56.3	59.8	66.8	74.2	78.3	81.1	85.4	88.2
10	0.259	70.798	0.154	52.4	54.5	57.8	60.2	63.7	70.8	78.4	82.8	85.8	90.4	93.6
11	0.045	74.843	0.148	56.5	58.6	61.9	64.2	67.7	74.8	82.7	87.2	90.4	95.4	98.7
12	-0.094	78.942	0.143	60.6	62.6	65.9	68.2	71.7	78.9	87	91.6	94.9	100.1	103.6
13	-0.157	83.222	0.138	64.5	66.6	69.9	72.2	75.9	83.2	91.4	96.2	99.6	104.9	108.5
14	-0.147	86.837	0.135	67.7	69.8	73.2	75.6	79.3	86.8	95.2	100	103.5	108.9	112.5
15	-0.057	88.965	0.134	69.3	71.5	75	77.5	81.3	89	97.4	102.3	105.7	111	114.7
16	0.115	89.706	0.134	69.4	71.7	75.4	78	81.9	89.7	98.2	103	106.4	111.6	115.1
17	0.346	89.544	0.135	68.6	71.1	74.9	77.6	81.6	89.5	98	102.7	106	110.9	114.3

L (Lambda; skewness), M (Mu; median) and S (Sigma; coefficient of variation).

The parameters of the ROC curves are shown in **Table 4**. The AUC for all groups is quite high and is close to 1. The model has a high capacity to discriminate between children and adolescents with low bone quality and those with normal bone quality. Sensitivity and specificity values in females are slightly higher than in males. However, the Youden Index indicates reflects 0.84 for both sexes and the appropriate cut-off point for males is ≤67.8 and for females is ≤63.7.

**Table 4:** Sensitivity and specificity values, area under the curve (AUC) and cut-off points for both sexes.

Parameters	Both sexes	Males	Females
AUC	0.956	0.965	0.971
IC%	0.946 to 0.964	0.957 to 0.972	0.957 to 0.985
Sensibilidad	0.60	0.61	0.71
Especificidad	0.96	0.96	0.98
Youden Index	0.81	0.84	0.84
Cut-off point	≤66.2	≤67.8	≤63.7

## Discussion

The initial objective of the study was to verify if there are differences in bone health with other geographic regions of the world. For this purpose, quantitative ultrasonography (QUS) using the Broadband Ultrasonic Attenuation (BUA) parameter was used in children and adolescents living in a high altitude region of Peru.

These results suggest that, although environmental conditions in high altitude regions present lower partial pressure of oxygen, colder temperatures and higher exposure to ultraviolet radiation (34). The general pattern of bone mass accumulation and its stabilization during adolescence seems to be consistent with that observed in other regions of the world and is associated with physical growth (13, 16, 35). This could indicate that, despite geographical and environmental variations, the process of bone development follows a relatively uniform course, reaching a plateau at similar ages. Furthermore, at advanced ages (16 and 17 yr), BUA values were relatively similar in relation to the Hungarian reference at low altitude (15), as in Peru at moderate altitude (17).

In fact, based on these findings, further studies comparing bone health at low, moderate and high altitude within the same population and/or geographic region need to be developed. This information could provide a solid basis for designing more precise and personalized interventions focused on bone disease prevention in these populations, taking into account environmental and genetic particularities that could influence bone health.

Given that patterns of bone mass accumulation appear to be relatively consistent in different geographic regions, but with slight discrepancies in absolute BUA values in relation to children and adolescents living at moderate altitude in Peru. The second objective of this study was to develop specific reference values for bone health in children and adolescents living at extreme altitude.

Indeed, in medical practice, percentiles are widely used to identify individuals whose conditions deviate from the normal distribution of their population (36). Thus, the proposed percentiles are an essential tool that would allow a more accurate and contextualized assessment of bone health in the pediatric population. The use of references could be useful to assess bone growth abnormalities in the prevention and follow-up of osteoporosis (37).

In the distribution of BUA percentiles, categories have been proposed as described by Gómez-Campos et al. (17), through which it is interpreted as: normal (>p10), low quality (p3 to p10) and very low quality (<p3). These categories thus facilitate a more detailed and useful classification for diagnosis and clinical follow-up.

In fact, the proposed percentiles allow a more precise interpretation adapted to the context, which is essential for early diagnosis and effective clinical follow-up in these specific populations. Thus low values of health can be recognized as osteoporosis and high values as normal growth among children and adolescents (38).

Consequently, bone health assessment, regardless of the measurement method used, is crucial to diagnose and monitor bone deterioration in children with chronic diseases or under medication (39). Therefore, it is essential to have assessment tools that reflect the particularities of each population group. In this regard, quantitative ultrasonography (QUS) presents itself as a viable option, as it allows a noninvasive, rapid and accessible assessment of bone quality. It is especially useful in environments with limited resources or in specific populations such as children and adolescents living in high altitude regions.

The third objective of the study sought to determine the specific cut-off point for the assessment of bone health (BUA) in this particular population. As a reference, the 10th percentile was adopted as the cut-off point according to some studies to categorize with low bone quality (5, 17). For this purpose, the model evaluated through ROC curves, evidenced a high discriminative power, especially in girls (BUA: ≤63.7). Where both AUC and specificity are slightly better than in boys (≤67.8). Although the sensitivity is moderate, so the high specificity and Youden’s index suggest that the selected cut-off point is effective in differentiating between children with and without low bone quality in pediatric high altitude populations.

Unlike previous studies, which have been limited to describing reference values (16, 22, 33), this approach allows a more effective and relevant application in clinical practice by developing specific percentiles and cut-off points.

In this context, percentiles and established cut-off points are tools that allow a more accurate assessment of the risk of bone deterioration. Especially in vulnerable populations (high altitude), such as those with chronic diseases or under medication. This contributes to better care and prevention of long-term complications.

The findings of this study suggest that quantitative ultrasonography (QUS), using the BUA parameter, is an effective tool for assessing bone health in children and adolescents. Given that in low-resource settings there is often a lack of awareness, guidelines, and adequate resources to manage pediatric bone disorders (39), QUS offers several advantages.

It is a safe, easy-to-use, radiation-free technique and its devices are portable. This makes it particularly suitable for assessing bone mineral status in children and adults (2, 40). This technique is particularly recommended in developing countries, where dual X-ray absorptiometry devices are often less accessible to the general population (41).

The study has several notable strengths. First, it focuses on a specific pediatric population residing in a high-altitude region in Peru. A population that has often been less studied compared to other geographic regions. Furthermore, the use of quantitative ultrasonography (QUS) as a method of bone health assessment is a remarkable approach, as it is a noninvasive, radiation-free and accessible technique, which makes it especially useful in areas with limited resources, such as high-altitude regions.

The study also has some limitations that should be considered when interpreting the results. The cross-sectional design of the study precludes establishing causal relationships and limits the observation of changes in bone health over time. In addition, the ability to generalize the findings to other populations is limited, given that the type of sampling was non-probabilistic.

Another limitation to consider is the possible variability in BUA measurements, since quantitative ultrasonography may be subject to both technical and physiological influences, which could affect the accuracy of the data. Therefore, it would be advisable that future studies use a reference method, such as dual-energy X-ray absorptiometry (DXA), to validate and corroborate these results. We also highlight that it was not possible to control for socioeconomic variables and lifestyles. Although, the schools investigated in this study are public schools. Those in Peru are often of middle/middle and middle/low socioeconomic status. Despite this, there is still a gap in the literature on these variables in young people living at high altitudes. It is therefore necessary to include them in future studies. As these studies can help mitigate substantial disparities in health behavior outcomes in children and adolescents (42).

The study demonstrated that small discrepancies in bone health (BUA) exist between children and adolescents from different geographic regions. These findings support the creation of specific references and cut-off points for bone health in the pediatric population of a high altitude region in Peru. The results suggest practical application in the assessment of bone health in school and epidemiological contexts.

## Data Availability

The raw data supporting the conclusions of this article will be made available by the corresponding author without undue reservation.
